# Impact of the COVID-19 Pandemic on Peer Support Services in the Veterans Health Administration: Lessons for Healthcare Organizations

**DOI:** 10.1007/s10488-026-01520-y

**Published:** 2026-08-29

**Authors:** Johanne Eliacin, Alexandria N. Miller, Marianne S. Matthias, Diana J. Burgess, Scott Patterson, Caitlin E. O’Connor, Angela L. Rollins

**Affiliations:** 1 Women’s Health Sciences Division, National Center for PTSD, Boston, United States; 2 VA Health Systems Research Center for Health Information and Communication, Richard L. Roudebush VA Medical Center, Indianapolis, United States; 3 Medical Center, Department of Psychiatry, Boston University, Boston, United States; 4 Women’s Health Sciences Division, National Center for PTSD, Boston, United States; 5 Department of Health Promotion Sciences, Mel and Enid Zuckerman College of Public Health, University of Arizona, Tuscon, United States; 6 VA Health Systems Research Center for Care Delivery and Outcomes Research, Minneapolis VA Health Care System, Minneapolis, United States; 7 Department of Medicine, University of Minnesota, Minneapolis, United States; 8 Department of Psychiatry Services, Richard L. Roudebush VA Medical Center, Indianapolis, United States; 9 Department of Psychiatry, Indiana University School of Medicine, Indianapolis, United States; 10 Department of Psychology, Indiana University Indianapolis, Indianapolis, United States

**Keywords:** Veterans, Peer specialists, COVID-19 pandemic, Adaptations, Implementation

## Abstract

To identify strategies that faciliate the implementation adaptation of peer support services to ensure continuity and effectiveness of mental health care during crisis or rapid organizational changes. This is a cross-sectional, qualitative study that was conducted during the COVID-19 pandemic with a purposeful sample of Veterans Health Administration (VHA) peer specialists and their supervisors from 11 facilities in a Veteran Integrated Service Network (VISN). Data collection and analysis were guided by the Consolidated Framework for Implementation Research (CFIR). Deductive and inductive thematic analysis approaches were used to analyze the data. VHA peer specialists employed various strategies that incorporated multiple CFIR domains to adapt to the COVID-19 restrictions and rapid organizational changes. Findings indicate that peer specialists as key individuals involved in delivering and implementing peer support services, as well as higher-level, outer setting factors such as patients’ needs drove these adaptations. Some strategies and CFIR domains were more influential than others in shaping these adaptations. Three key strategies emerged as potentially useful in future public health crises and rapid organizational changes to expand the capacity of peer specialists’ role to meet patients’ healthcare needs: 1) adopting a work culture that is receptive to change (learning climate), 2) promoting collaboration (network and collaboration), and 3) fostering peer specialists’ self-efficacy (characteristics of the individual). During times of crisis, foundational organizational practices, such as change management techniques and transformational leadership styles, can help support service delivery adaptations and improve care delivery to patients.

## Highlights

1. Similar to other aspects of health care and healthcare organizations, VHA peer support services experienced disruptions during the COVID-19 pandemic that included work stoppage, communication challenges, and changing work dynamics.
2. In response to the pandemic, VHA peer specialists made several adaptations that facilitated continuity of peer support services delivery. These adaptations demonstrated the services’ agility, resilience, creativity, and high potential to make meaningful contributions to VHA as a learning health system in times of rapid change.
3. During times of crisis or rapid organizational changes, foundational organizational practices, such as change management techniques and transformational leadership styles, can help support services delivery adaptations and improve care delivery to patients.

## Introduction

The novel coronavirus (COVID-19) pandemic had profound negative mental health impacts on individuals and communities in the United States (U.S.) (Sousa et al., 2021; Pfefferbaum & North, 2020). Early in the pandemic, individuals diagnosed with COVID-19 developed increased risk of psychological distress (Jafri et al., 2022). During the height of the pandemic, persistent stress from social isolation, fear of exposure to the disease, and financial strains led to a host of mental health problems (Brooks et al., 2020; Sousa et al., 2021; Wang et al., 2020). The prevalence of depression, anxiety symptoms, emotional disturbance, substance use, and suicidal behavior increased significantly (Hossain et al., 2020; Ngo et al., 2025). Patients with existing mental health conditions saw exacerbation of psychiatric symptoms (Na et al., 2022) and individuals from socioeconomically vulnerable communities experienced disproportionately worse mental health outcomes and higher mental health burden compared to their counterparts (Lee & Singh, 2021; Matthias et al., 2022; McKnight-Eily et al., 2021; Thomeer et al., 2023). Among U.S. Veterans, a vulnerable population with high rates of comorbid mental health conditions (Beech et al., 2021; Hadlandsmyth et al., 2024), COVID-19 related stress and worsening symptoms of post-traumatic stress, depression, and anxiety were common (Hill et al., 2023; McLean et al., 2022; Pedersen et al., 2021, 2023). Further, a systematic review of 23 studies, most of which were from the U.S., indicated that compared to non-Veterans, Veterans experienced more mental health problems during the pandemic (Li et al., 2023).

Additionally, efforts to contain the disease early in the pandemic, during the primary U.S. surges from March 2020 to October 2020, led to massive disruptions in mental services delivery across many health organizations (Holland et al., 2021; Zhu et al., 2022). These disruptions included surges and fluctuations in demand for mental health visits, accompanied by decreased access to mental health treatment due to cancellation of in-person visits, reallocation of staff to COVID-19 related care, and the shift to tele-mental health services delivery (Holland et al., 2021; Titov et al., 2020). Similar to other health organizations, the Veterans Health Administration (VHA), which is the largest integrated healthcare system in the U.S., experienced significant challenges in meeting demands for mental health services (Leung et al., 2023; Tran et al., 2021). For example, McGuire and colleagues reported substantial disruptions in inpatient mental health services in the VHA, where patients were less likely to receive individual therapy, group therapy, and peer support services following the onset of the pandemic (McGuire et al., 2021). Other studies also documented disruptions in the delivery of evidence-based psychotherapies and fewer mental healthcare visits (Cully et al., 2024).

Given these abrupt and significant organizational changes, we sought to investigate how the VHA leveraged the peer specialist (PS) mental health profession to meet the mental health needs of Veterans during the primary surges of COVID-19, starting from March 2020. Prior to the pandemic, the VHA actively expanded its PS workforce to better support Veteran patients throughout their healthcare journey. This effort included legislative measures, such as President Obama’s Executive Order 1365 in 2012 that mandated the Department of Veteran Affairs to hire and train 800 PS before 2014 (Executive Order No. 13,625, 2012). In addition to the VHA, community mental health organizations have a long history of seeking to enhance the mental health workforce to meet increasing mental health needs by training paraprofessionals such as PS (Mowbray et al., 1993; Smit et al., 2023; Stack et al., 2022). While PS are not clinicians, they are mental healthcare professionals who use their lived experiences and formal training to support others in their recovery or wellness goals (Center for Mental Health Services, 2024). PS are often the first point-of-contact for behavioral services to individuals in various health and social settings such as hospitals, community, substance use programs, shelters, and crisis teams (Center for Mental Health Services, 2024; Stack et al., 2022). They provide support, treatment, and resource navigation. In VHA, PS are Veterans with lived mental health experience, who are trained and certified to support patients through their healthcare journey (Chinman et al., 2012, 2017, 2019). They work as part of a clinical team, typically in specialty mental health settings such as outpatient mental health clinics, inpatient and residential programs, vocational rehabilitation services, Veteran justice programs, substance use and homeless programs, where they support clinicians and patients. VHA PS often serve as liaisons between patients and the treatment team. They function as role models for patients, facilitate recovery-oriented and evidence-based psychosocial interventions, such as social skills training for patients with severe mental illness, and provide social, logistical, and emotional support to patients to engage them in care (Chinman et al., 2014, 2019; Davidson et al., 2006; Eliacin et al., 2022).

While the number of PS positions in VHA has increased rapidly since 2012 (Eliacin et al., 2023), their integration in clinical services has faced some challenges. Several studies have documented barriers to implementing peer support services in VHA and other healthcare systems, which include lack of clarity of PS role, stigma and distrust of PS based on their identity as mental health consumers, cultural resistance to the peer support services care model, existing workflows, and power dynamics between peer support staff and other team members (Chinman et al., 2010, 2015, 2021; Eliacin et al., 2020; Mutschler et al., 2022; Peeples et al., 2023). Many of these barriers not only hinder PS integration within healthcare systems but also undermine their patient work and overall job satisfaction. For example, many PS experience inadequate training, supervision, logistical support, and compensation, which contribute to burnout and low job satisfaction. Additionally, PS are sometimes excluded from clinical team meetings and denied access to relevant patient information, which limits their ability to function effectively. (Collier et al., 2024; Shepardson et al., 2019; Vandewalle et al., 2016).

Moreover, prior to the pandemic, PS delivered services primarily in-person in VHA clinics and in the community through their outreach efforts to Veterans and community organizations. However, during the pandemic, the VHA significantly expanded the use of telehealth services delivery to meet demand for care and to reduce risk of infection for both patients and clinicians (Connolly et al., 2021; Partnered Evidence-based Policy Resource Center, 2023). This shift in mental health service delivery upended many aspects of peer support services, while also creating opportunities for engaging PS in other capacities.

The primary objective of this study was to identify strategies that could inform future service adaptations to maintain or enhance peer support services delivery in times of crisis or rapid organizational changes to meet patients’ mental health needs. Specifically, we aimed to document how the pandemic impacted VHA PS and peer support services. Given that health crises and rapid organizational changes can arise unexpectedly, we also sought to examine how VHA PS navigated the pandemic and its associated challenges. To develop a deeper understanding of peer support services adaptations, we also included the perspectives of PS supervisors. To guide our data collection and analysis, we used elements from the original version of the Consolidated Framework for Implementation Research (CFIR) (Damschroder et al., 2009), which offers a comprehensive framework that facilitates systematic assessment of factors that influence implementation of evidence-based programs or interventions.

## Methods

This was a qualitative study with a purposeful sample of PS and peer support supervisors from 11 VHA facilities in Veterans Integrated Service Network (VISN) 10. VISN 10 is a regional system that includes VA Medical Centers and Community Based Outpatient Clinics in Michigan, Ohio/Northern Kentucky, and Indiana. PS supervisors, hereafter, supervisors, in most VHA settings are health care professionals, such as social workers, psychologists, and PS who provide training and clinical supervision to peer support staff within a clinical team or a facility. Participation in the study was open to any peer and supervisor in the VISN 10. Participants were recruited from 8/11/2020 to 2/08/2021 through direct outreach by email, presentations at regional team meetings of PS, and snowball sampling methods, asking enrolled participants to refer other potential participants to the study (Noy, 2009). Participants provided informed consent prior to study participation. Per VHA policies, VHA employees were not eligible for compensation or incentives for their participation in the study. The study was approved by the Richard L. Roudebush VA Medical Center Research & Development Committee and theIndiana University Institutional Review Board.

We conducted semi-structured interviews with participants over the phone or via VA Microsoft Teams. Interviews were conducted by trained qualitative interviewers, lasting from 30 to 60 min, and were recorded, transcribed, and de-identified for analysis. Guided by the original version of CFIR (Damschroder et al., 2009), interviews focused on identifying adaptations to implementation of peer support services made in response to the pandemic that facilitated continuity of peer services delivery. In this study, we explored factors from four CFIR domains characterizing drivers, contexts, and strategies for implementing adaptations: *Inner setting* (climate and organizational structures that prompted and/or facilitated adaptations), *Outer setting* (broader organizational system and policies), *Characteristics of individuals involved* (PS’ training, skill levels), and *Implementation process* (strategies and tools employed for adoption). Examples of questions included: *Tell me how COVID-19 has affected your work. Tell me about the changes that were made to your peer support services during the COVID-19 pandemic. How did the process of making these changes go? What worked well and what did not? What service gaps do you see in your clinic/setting at this time? If there is a resurgence of COVID-19…, how could the VHA, specifically mental health services, be better prepared to meet Veterans’ mental health needs?* In addition, we collected demographic data from participants.

### Data Analysis

A team of 8 analysts, which included the lead author (JE), a clinical psychologist and qualitative methodologist, the senior author, (AR), a clinical psychologist and implementation scientist ), and 6 trained research assistants, analyzed the data using deductive and inductive thematic approaches (Braun & Clarke, 2006). We first used a deductive approach. We developed a codebook consisting of pre-identified domains and elements from CFIR to organize the data, such as *inner setting and adaptations made*. The analytical team independently coded a subset of transcripts (n=5, 18%) and iteratively revised the coding book to maximize consistency. To ensure analytic rigor, most of the transcripts (69%) were coded independently by at least two coders, with the team collaboratively finalizing the coding for all transcripts. Discrepancies were resolved by consensus. Then, we conducted inductive, axial coding (Saldaña, 2013). This involved combining excerpts from similar or related codes within each CFIR domain and conducting additional analysis using sub-codes that emerged from the data. The subcodes were iteratively refined and added to the code book. This process facilitated identification of new categories and themes, as well as connections between different sections of the data. Interview transcripts from PS and supervisors were coded independently using the same coding book. We summarized the findings and organized them using a templated matrix consisting of participating roles (PS and supervisors), sites, themes, and CFIR domains. Then, the study team identified the presence or absence of themes (e.g., implementation adaptations made) and CFIR domains and elements (e.g., external work policy) at each site. We compared summaries based on participant roles to identify themes and subthemes that are similar, complementary, or different. However, our goal was to provide an integrated, comprehensive view of PS experiences using both PS and supervisors’ perspectives. NVIVO qualitative data analysis software was used to manage the data (Lumivero, 2018).

## Results

### Participants’ Characteristics

As shown in Table 1, 18 peers and 10 supervisors from 11 VHA facilities in Ohio, Indiana, and Michigan participated in the study. The range of participants per site was 0 to 4 for PS and 0 to 3 for supervisors. Participants (PS and supervisors) had an average of 5.26 years of tenure in their position at the VA. Most were male (64%), White (54%), and over age 50 (71%). Women PS were oversampled in this study. 39% of the PS participants were women compared to 19% to 20% of the VHA peer workforce in 2020. (Department of Veterans Affairs, 2022). Participants worked in diverse VA settings, including outpatient mental health clinics, inpatient, residential treatment, and programs for unhoused Veterans.

**Table 1:** Participants’ characteristics

	Peer specialist (*n* = 18)		Supervisor (*n*=10)		Total (*n* = 28)	
	N	%	N	%	N	%
**Tenure**						
Average number of years in position, Standard Deviation (X, SD)	5.08 (2.87)	n/a	5.58 (4.06)	n/a	5.26 (3.28)	n/a
Average number of years in position at facility, Standard Deviation (X, SD)	4.72 (2.75)	n/a	6.33 (4.12)	n/a	5.29 (3.32)	n/a
Average number of years at VA, Standard Deviation (X, SD)	8.17 (4.96)	n/a	10.58 (5.48)	n/a	9.03 (5.18)	n/a
**Sex**						
Male	11	61	4	40	15	64
Female	7	39	6	60	13	46
**Ethnicity**						
Hispanic	0	0	2	20	2	7
Non-Hispanic	18	100	8	80	26	93
**Race**						
Black/African American	9	50	3	30	12	43
White	9	50	6	60	15	54
Unknown	0	0	1	10	1	4
**Age**						
30–39	0	0	2	20	2	7
40–49	4	22	2	20	6	21
50–59	5	28	4	40	9	32
60–69	9	50	2	20	11	39
**Education**						
Some college	12	67	0	0	12	43
Four years of college	4	22	1	10	5	18
More than four years of college	2	11	9	90	11	39

### Disruptions and Challenges

In what follows, we first present negative impacts of COVID-19 restrictions on PS and their work. Then, we present adaptations made to facilitate PS work and their impact on patient care. The pandemic disrupted many aspects of peer support services, created communication challenges, and negatively affected work dynamics between PS, supervisors, and work teams. Below, we highlight the most salient themes that emerged from participant interviews. Supportive quotes are included in Table 2.

**Table 2:** Disruptions and challenges

Themes	Exemplar quotes
Technology barriers	*Our skills are being put to the test because we went from Skype to Teams. … Some people are not as savvy as others. …We may get on a call, and nobody knows where the mute button is…. –Peer Specialist* *It’s not easy for someone who hardly accesses the internet… we’re telling them to click on links. They don’t really understand that.—Peer Specialist* *Because I work with primarily the SMI [Severe mental illness] population, the success of doing video with the SMI population is limited because of the severe paranoia, feeling that they’re going to be recorded.—Peer Specialist* *I get frustrated with the Zoom because … people are talking over each other. If I’m sitting in a room with you, I can tell you’re going to say something. It’s hard. A participant got mad because he didn’t get to ask a question in the budget group yesterday. He said he’s raising his hand, but I can only see so many people here. I didn’t see anybody raise their hand. – Peer Specialist* *The peers that I had didn’t have any telephone experience, putting in telephone codes, and progress notes, and phone codes. (laughter) It was all of a sudden very different kind of experience because, you know, most of the peers’ interaction is face to face.” -Supervisor*
Disruption in services	*I used to do a lot of community outreach in my role. … [COVID-19] really harmed my ability to network like I used to… they’re not even thinking about me anymore… It hurts our partnerships. … –Supervisor* *We have 3 providers who work the mid shift tour, and with COVID restrictions, they’re now on the day shift. So, there are a lot of Veterans who now don’t receive our services because they were only able to come in the evening. You know, right now during COVID, we don’t [provide] evening services. They could if the staff hadn’t been moved to the day shift. So, while we’re under COVID restrictions, we’re, everybody’s on the day shift. -Supervisor* *Because they [Veterans] are still being in contact with the Department of Corrections, the challenge I find is a lot of them have not been registered for services. And because of their incarceration, and of the void or that liaison, intake liaison, social worker,… getting the documents for them now is more challenging because a lot of places are closed. – Peer Specialist* *"So, it’s very difficult. That was a big part of my job, which was going out and saying this is what we do… I was really good at recruiting and letting people know and not only that, I talked about the mental health clinic and all the services they provide.- Peer Specialist*
Challenging work dynamics	*As an employee it’s driving me crazy because I sit here in an office, look at a brick wall and there’s nobody to talk to. Everybody’s got their door shut. I can’t do a lot of the stuff that I used to do. …I’m just frustrated with having to sit here. – Peer Specialist* *This is probably the one thing I got most frustrated about during the whole pandemic was people that were doing very similar jobs to us got to go home and work from home. … however, they said we were essential staff. …they pulled one peer to do sewing for masks. …So, I think, [they need to] utilize us to our full potential not saying, oh, well, they’re just peer support. – Peer Specialist* *I didn’t even know we were having groups until my supervisor brought it to my attention because I was off that email chain. He said they’re starting groups next week. No one told me… I haven’t gotten an email in two months about what we’re doing. – Peer Specialist* *“ I miss the comradery or the ability to have like a preceptor or somebody who’s, you know, working shoulder to shoulder, helping me to understand my role.” -Supervisor* *“Well, I would say, I think, you know, just the screen time drain. You know, I can remember just being so exhausted. You know, shifting to sitting, not even, like feeling like I couldn’t hardly get up to go to the bathroom in between things because there’s not a break…So, just adjusting to all the screen time, all the sitting and not moving as much, having to be more intentional about moving and stretching, and you know, walking the stairs, or getting outside.”- Supervisor* *Here’s what’s happened since the pandemic that I noticed, that says a lot. When we switched over to virtual, some supervisors went to this thing, “I got to make sure they’re productive.” They had each staff member meet with them at the end of the day. “Who did you see? How many phone calls did you make? It’s like there was a complete lack of trust. It’s like the peers have to defend themselves. It sets up this kind of difficult interaction, where it’s not productive. –Supervisor*

#### Technology Barriers

 Prior to the pandemic, most peer support services were delivered in-person. Therefore, participants (PS and supervisors) reported that the swift transition from in-person to telehealth services delivery (phone, video connect) was a major challenge for many PS who had limited computer skills. PS in particular expressed frustration with the logistical barriers for accessing and learning approved platforms for telehealth. They also reported missing the camaraderie of in-person interactions with their Veteran patients and found the hours spent conducting phone wellness outreach to be monotonous and burdensome. Participants also noted that the transition to telehealth services delivery was impacted by Veteran patients’ barriers that included lack of internet connectivity, limited access to communication devices, and limited technology skills. For example, some patients did not have enough minutes on their mobile phones, or had their phones broken or stolen, which affected their access to mental health services.

#### Disruptions in Services

 As noted above, an effective and key component of peer support service is outreach to Veterans and community organizations to engage Veterans in VHA care and to facilitate Veterans’ access to health and social services such as housing, financial, and social support. In addition to halting PS in-person services delivery, the pandemic also disrupted VHA interagency collaborations and PS community outreach activities due to physical distancing, community partners’ changing priorities, and discontinued or reduced community activities. Participants shared that these disruptions limited PS work and their ability to support Veteran patients. The disruptions also abruptly paused or terminated valuable long-term organizational partnerships that benefited Veterans.

#### Challenging Work Dynamics

PS discussed barriers as they adjusted to remote work, and challenges related to what they perceived was unfair implementation of telework policies that were unfavorable to PS, reduced autonomy to perform their work, and led to ineffective team communication that left them feeling excluded from their work team. Some PS noted that they were denied or were the last ones to be approved for remote work accommodation during the acute phase of the pandemic, despite having high risks of infections, while other team members, such as clinicians, were granted remote work privileges. Additionally, PS discussed that they felt they held less power and status on their teams, which may have contributed to being left out of team decision-making processes and relevant information about clinical services. PS also discussed that the transition from in-person to remote work led some supervisors to micromanage their daily tasks, where they needed to account for every hour and provide evidence of productivity, when previously they were afforded ample autonomy to perform their work. Some supervisors also corroborated these findings. Many participants shared that this change in management style reflected distrust and negative perceptions of PS. On a personal level, some PS reported feelings of loneliness and boredom from remote work as well as from physical distancing at work and unscheduled work hours. Additionally, both PS and supervisors commented that it was a challenge to maintain effective team communication and workflow as many teams were overwhelmed with high volume of new information from multiple sources and rapidly changing policy landscape.

### Context for Peer Support Services Adaptations

In response to the COVID-19 pandemic, VHA PS made several adaptations to maintain continuity of peer support services delivery and to address emerging social and mental healthcare needs of Veterans. Because peer services or specific duties differ widely across settings, we do not include the intervention CFIR domain in this report. Instead, we focus on documenting the sources and contexts of adaptations made, which fall under CFIR domains of inner and outer settings. Adaptations varied across sites and were influenced by different factors from both inner and outer settings. Below, we describe these adaptations and characterize them by CFIR domains. For brevity, we highlight adaptations that were most salient in participants’ (PS and supervisor) interviews. We also summarize the number of sites with similar adaptations, themes that emerged within each domain, and provide exemplary quotes in Table 3.

**Table 3:** CFIR Domains and Adaptations Made to Maintain or Enhance Peer Services Delivery

	Presence or absence of themes across sites, N=11 sites	Examples of adaptations made/ lack of adaptations	Exemplar quotes
**Outer setting**	**Veterans’ Needs**(*i.e., Adaptations that were made because of patients’ needs*)	• Shifted from in-person to virtual services delivery • Developed new initiatives to meet Veterans’ needs, such as access to new apartments or hotels for Veterans experiencing homelessness • Modified group formats to reach more individuals. For example, instead of holding a single 60-min in-person group with 10 participants, they split it into two 30-min groups with 5 participants each to meet social distancing guidelines. This allowed all 10 participants to attend rather than cancelling the group entirely • Delivered services to new Veteran populations. For example, at one site, when their financial literacy outpatient in-person groups were cancelled, PS started offering the groups to patients in residential and in-patient settings • Transitioned from group classes to one-on-one phone and video meetings to maintain patient engagement and services delivery	Groups were cancelled, then they were brought back, but they were limited to 10 [Veterans] based on the size of the room to allow them physical distancing. …if we had more than 10 show up …, we would do 2 half hour sessions. …I had to be flexible. -Peer Specialist What they did with that check-in group (new group) was [to create] a place where people could come in and talk about anything…they didn’t have to talk about recovery…or mental health issues… And what I heard from Veterans were things like this really saved me. This was my chance to really just talk to people cause otherwise I’m stuck in my apartment all day. -Supervisor
	**External policy & incentives**	• Policies established for peer specialists to disseminate COVID-19 guidelines to Veterans for public health promotion • Peer specialists assigned to conduct COVID-19 screening at their facility • National and facility-level policies as well as CDC guidelines to reduce risks of COVID-19 such as conducting groups outside in open-air to facilitate in-person attendance	They’re just starting to do face-to-face groups in the residential programs. And again, they’ve been tested for COVID before they’re brought in. We’re really only trying to take people who are local and then everybody of course has to wear masks. And we have distancing. Supervisor Our homeless program office (VA Central Office) when all of this started, they were really great with the flexibilities that they allowed …. So, right now we probably have 90 of flex placements… and this is really just to stabilize folks during the shutdown… So, it’s 100% a COVID response this thing that’s brand new. Supervisor
**Inner setting**	**Tension for change **	• Increased delivery of peer support services to meet Veterans’ mental health needs and decrease wait- time for engagement in the clinics. For example, peer specialists were casting a wide net, to reach out to Veterans for wellness visits and peer support services • Peer specialists reached out to new settings to deliver services to new Veteran populations, such as rural Veterans and those not connected to VA services because of decline in in-person and outpatient visits • Adopted telehealth peer support services delivery due to cancellation of in-person visit and demands for productivity	I had staff here who did not want to connect virtually. …And there was really no pushing on my part that could get them to move. Their supervisors weren’t trying to push them…So, when this happened, I immediately sent out an email saying, we need to create a VA virtual university that offered groups to Veterans. … And they got shutdown, they could no longer go into the community. They saw this as an opportunity to keep their (productivity) numbers up. So, they came to me and said, every one of our homeless people will do a group… we started out with like 12 but then like as they sort of mandated you are going to do groups, we started to fill out into 30…. And we grew, and we grew to 50. So, the idea came from the need. -Supervisor We’re not doing the budgeting and credit class [in outpatient settings]…Because that’s not going on, the peer specialist on the PTSD ward and I are teaching it via Zoom to all three residential wards. -Peer Specialist
	**Learning climate**	• Some sites encouraged peer specialists to use their skills in variety of ways and accommodated their comfort level with technology • Sites provided training and increased supervision to support transition to telehealth services • Sites encouraged peer specialists to complete new training, expand their skills, and obtain online certificates in motivational interviewing, Whole Health, etc	I have some older peers, who struggle with technology… So, what I’ve learned is not to try to force their comfort. I try to utilize them in other ways to address other situations, making phone calls, doing things like that, that they’re more comfortable with. – Supervisor COVID kind of afforded us more time to do training. … in the office students needed stuff to do, so I had the one intern who did that MI support. And then I had another Practicum student, who helped them (peer specialists) kind of reformulate how we do a group facilitation. How we were going to do it virtually.” -Supervisor
	**Networks & communications**	• Sites developed new networks across facilities to share ideas and gain support. For example, peer specialists at different facilities collaborated to co-lead a new group. Supervisors sought guidance and consultation from others at different settings • Increased community outreach and collaborations to meet emerging Veterans’ needs • Increased frequency of supervision and developed new strategies to facilitate team communication • Created a VA virtual university to facilitate training of new peer specialists	So, the first thing we did was we created about 50 offerings throughout the week. And these were done by peers, psychologists, social workers. You know, there was all this extra time, so, the idea was I should pick something up. And the idea wasn’t that we expected any of these groups to be filled." -Supervisor I have been able to connect with people further away…I just got a request from a peer in [CITY/STATE 2] which is many miles away from me, to help him do an introduction to whole health with people that he has through VVC (video-conference). And we would have never thought of that pre Corona…so that’s another change. -Peer Specialist
	**Leadership engagement**	• Provided support and facilitated manageable workloads • Ensured telehealth and remote work privileges for peer specialists • Protected peer role and increased their visibility	Our chief has been very supportive of whatever we need to do to make this work… there’s some VA’s where they fight you tooth and nail to get a day of telework, whatever… we just kind of worked it all out. And our chief said, do what you need to do. You know, like let’s keep serving the Veterans.—supervisor
**Readiness for implementation**	**Available resources**	• Distributed phones and iPads to Veterans to facilitate telehealth services • Expanded VA telehealth mental health services • Placed unhoused Veterans in hotels while they waited for placement • Conducted housing search virtually with Veterans	I was pretty was able to get iPads for almost all Veterans that I meet with. That was so awesome. I’m so happy about that because not only could they meet with me and stay in contact with me, but they could also stay in contact with their providers. So that really helped them. -Peer Specialist We were working with our community partners as far as registration, getting the homeless registered at, we were working with them through FAX machines, telephone calls, you know, stuff like that. -Peer Specialist
	**Access to knowledge & information**	• Access to regional network of peers and supervisors as well as VA infrastructure that facilitated exchange of information • Drew and learned from peer specialists’ lived experiences and understanding of Veterans’ social contexts provided access to information about Veterans’ unmet needs • Capitalized on the availability of online training resources • Utilized VA telehealth technology and resources	What I love about being virtual is, you know, sometimes you can’t always get a person in your office, right, to show them certain stuff. So, now, if you can get them on video, you can share your screen, and they could show them like, look at this unit. Or this is how you do the search. This is how you do a housing search. And kind of really walk them through, even to a point where, you know, even when we did do it in person, and we could show them how to do a housing search. But now to say, okay, now share your screen. Let me see you do it. That’s different.” -Supervisor
**Characteristics of individuals**	**Individual Characteristics**	• Peer specialists served as agents of change, created new groups, community partnerships, and services, such as food pantries to meet Veterans’ needs • Peer specialists learned new technologies to do their work and facilitate Veterans’ telehealth visits • Many peer specialists worked weekends and after hours to support Veterans (e.g., deliver goods, complete extra paperwork, and conduct outreach) • Peer specialists used their lived experiences and knowledge of Veterans’ social contexts to identify and address unmet social needs	“I’m more than willing to help out in some way, something that’s going to benefit the VA or a Veteran, as long as there’s an open area in my schedule. I just stay busy.” -Peer specialist The toilet paper shortage that was going on and paper towels, we started having Veterans calling like, I can’t get this stuff… can I come down there and get some? or can you bring me someone we were more like, please don’t come down here cause then there again, you got to catch the bus…And so, the peers would start contacting me [asking], if Veterans need certain stuff, can we drop it off? And then, I was like, you know, that’s a good idea. So, we talked about how, what’s the best way to do it? So, make sure you have the appropriate PPE, you know, you do not go in the home, you know, keeping social distance.....and then from there, from us doing our own in-house pantry, we started seeing people with food situations … And so, the peer specialist had asked the person who was running the pantry … to create some food boxes, and we’ll deliver them. -Supervisor

#### Outer Settings

Peer support services adaptations were driven by both ***patients’ needs and external policies***. PS and supervisors emphasized that meeting Veterans’ needs was the most important driver for program adaptations. Indeed, 10 of the 11 sites reported making peer support services changes to meet Veterans’ mental health needs. Examples of adaptations made included expanding peer support services telehealth offerings; developing new programs to support Veterans’ emerging mental health needs, such as experiences of social isolation and loneliness; modifying group structures to increase access; and extending peer support services to new subgroups of Veterans, such as patients in inpatient and residential settings, once outpatient healthcare visits were cancelled. Participants (PS and supervisors) also reported that VHA national and facility-specific COVID-19 guidelines facilitated some peer support services adaptations. Decisions to shift to telehealth peer support services delivery and the implementation of that policy varied based on locality and facility. Further, PS at some facilities were re-assigned to contribute to COVID-19 related duties, such as disseminating information about US Centers for Disease Control COVID-19 guidelines to Veterans and enforcing physical distancing and masking policies.

#### Inner Setting

Several CFIR inner setting elements influenced PS role and services adaptations. However, inner elements such as Sect. “Tension for Change” and Sect. “Learning Climate” were the most frequently reported elements, followed by Sect. “Networks and Communication***”*** while Sect. “Leadership Engagement” was reported less often.

#### Tension for Change

This element was prominent amongst most sites (*n*=9). To illustrate, participants (PS and supervisors) explained that prior to the pandemic, many PS were reluctant to adopt telehealth despite VHA’s pioneering work in telehealth services delivery, supervisors’ recommendations, and national implementation push. However, once in-person visits were cancelled, some PS felt pressured to maintain work productivity. Only then and “*out of necessity*,” did they adopt telehealth (video) peer support services delivery. However, some PS struggled with the technology and adjusted to this pressure in different ways. For example, some PS cancelled group classes and offered instead short individual phone meeting sessions, which led to increased use of phone communication and undermined efforts to increase video telehealth visits.

#### Learning Climate

Most sites (*n*=7) benefited from their learning climate to inspire and facilitate adaptations. Learning climate is characterized as a program’s or a setting’s flexibility and openness to new ideas and change. In these environments, PS felt comfortable sharing ideas with supervisors on how to best meet Veterans’ needs and were involved in leading new initiatives. Supervisors at those sites also demonstrated flexibility in their approaches to supporting PS with technological challenges. While there was pressure to shift to telehealth services, some supervisors adapted by matching PS to services based on their skillsets and comfort level with learning new technologies. A supervisor explained that PS at their site were uncomfortable learning new software to deliver virtual peer support services. As a result, they were assigned to make brief wellness check calls to Veterans instead of delivering virtual groups. Additionally, because of in-person visit cancellations, PS had unscheduled time. At some sites, supervisors encouraged them to take on new online training courses, such as motivational interviewing or to reconfigure PS-led groups to maximize their effectiveness and enhance the quality of peer support services, while others were not offered these opportunities.

#### Networks and Communication

During the pandemic, several sites (*n*=6) expanded their networks to identify new resources or to develop alternative strategies to meet Veterans’ needs. An example of such an adaptation was PS and supervisors moving from working in silos within their individual sites to collaborating across VHA departments and facilities, sometimes across states, to co-facilitate PS-led groups or develop new peer support services. Moreover, PS expanded their outreach to new community organizations to identify resources to meet Veterans’ unmet social needs. Supervisors also adapted by expanding their network to include other supervisors across VHA facilities to share ideas on navigating COVID-19 challenges, seeking support, and developing new resources to meet PS and Veterans’ needs. For example, a supervisor described reaching out to colleagues at other facilities to identify billing codes for telehealth peer support services. Moreover, supervisors developed new communication strategies to enhance information sharing and to facilitate regular team feedback, maintain trust, transparency, and team efficacy, especially in the context of rapid policy changes and daily influx of COVID-19 related information. These strategies included increased frequency of supervision, taking meeting minutes for later dissemination, and sharing important team announcements via a centralized system (e.g., from one delegated person, at a regular time, using written format).

#### Leadership Engagement

Discussions about leadership engagement were limited in content and centered on immediate supervisors’ activities. Only 5 sites had participants who specifically described adaptations facilitated by leadership engagement. For example, participants described how supervisors facilitated manageable workloads for PS and access to telework privileges. PS and supervisors also reported that supervisors protected PS roles amid some facilities’ pressure to reassign peers to perform duties outside their scope of practice, such as conducting hospital-based COVID-19 screening. PS from homelessness programs also discussed leadership engagement at the regional and national level that provided direction for their work, including placing Veterans experiencing homelessness in hotels and conducting virtual home tours to support their housing search.

### Process for Implementation

#### Available Resources

Use of available resources was a salient theme across most sites (*n*=10) as participants credited adaptations made during the pandemic, in part, to the agility of the VHA as a learning healthcare system to rapidly shift resources to new services and meet Veterans’ needs. Some sites were already providing telehealth peer support services prior to the pandemic and were well poised to expand those services. Peer support services also capitalized on the wealth of VHA resources to make rapid adaptations and maintain mental health services delivery. Some VHA sites distributed communication devices (e.g., tablets and phones) to Veterans to facilitate telehealth visits and utilized occupational therapy services to train Veterans on how to use the technology. Audiology departments provided clear masks to facilitate PS-led social skills groups for Veterans with mental illness. Moreover, PS referred Veterans to VA virtual tools and online resources, such as PTSD and sleep apps for self-care management, while they waited for an available mental healthcare clinician.

#### Access to Knowledge and Information

Participants (PS and supervisors) from most sites (*n*=7) also had access to knowledge and information to evaluate and modify their practices as needed. They benefited from the infrastructure of a regional network of PS and supervisors to share ideas. Furthermore, PS served as primary points of contact for many Veterans and as liaisons between patients and clinicians and the healthcare system. As such, they facilitated valuable exchange of information that benefited Veterans’ health care. PS were able to identify Veterans’ needs (e.g., unmet social needs), obtain feedback from Veterans on adaptations made (e.g., difficulties with technology), and address these challenges with the support of their supervisors and clinical team members. Participants also shared that they rapidly piloted several adaptations and made iterative changes as needed. An example includes restructuring groups facilitated by PS to different durations, formats, and group sizes to facilitate access to care. These adaptations facilitated effective response to rapid procedural and policy changes and helped maintain and improve mental health services delivery.

#### Characteristics of Individuals

In this section, we describe PS roles and characteristics as active agents of implementation and deliverers of peer support services. Some sites (*n*=6) capitalized on PS lived experiences and shared identities with Veteran patients to address COVID-19 related challenges and improve peer support services. These supervisors actively solicited and encouraged PS input in programs’ decisions, and maintained PS autonomy, which led to creative program developments. In fact, both supervisors and PS described several new activities that were initiated by PS. For example, a PS described using his story of incarceration and negative mental health impacts of isolation to encourage leadership at a residential program to change their policy of restricting residents to their rooms all day as a measure of COVID-19 risk reduction. Another discussed how she partnered with a food pantry to deliver food to the homes of older Veterans in need. Other PS had opportunities to take on new administrative responsibilities such as creating reports and leading new groups. In brief, some programs supported PS self-efficacy and emphasized their values as agents of change.

## Discussion

To inform future crisis or rapid organizational changes needed in VHA, we sought to describe how PS and supervisors in the VHA perceived the impact of COVID-19 restrictions on peer support services and the adaptations they made to meet the mental healthcare needs of patients. In so doing, we examined PS and supervisors’ experiences of the early COVID-19 pandemic period and how they adapted peer support services to meet the increasing mental health needs of Veteran patients.

Our findings indicate that PS and peer support services were negatively impacted by the COVID-19 pandemic and its aftermath. First, many aspects of peer support services were paused or terminated, especially services that were delivered in-person and that relied on partnerships with community organizations. This is consistent with reports about widespread disruptions in health and mental health services delivery across various healthcare organizations during that period (Dinkeloo & Luse, 2022; McBain et al., 2023; McGuire et al., 2021). Additionally, the unique stress and work demand of the pandemic exposed existing power dynamics and system challenges that limited PS’s ability to adapt their delivery of peer support services. PS echoed many well-known implementation barriers associated with the peer support care model across various healthcare organizations, such as lack of clarity of the PS role and incomplete integration of PS in work teams (Cooper et al., 2024; Eliacin et al., 2020; Hamilton et al., 2015; Moll et al., 2009).

Our findings also show that PS employed a wide range of strategies that incorporated multiple CFIR domains to adapt to COVID-19 challenges and rapid organizational changes. First, they demonstrate that first-line health care professionals and services deliverers, such as PS, as well as higher-level, outer setting factors such as patients’ needs drove these adaptations. Moreover, we found that some strategies and CFIR domains were more influential than others in shaping these adaptations. Participants (supervisors and PS’) discussion of peer support services adaptations most frequently included CFIR elements of patients’ needs, available resources, and tension for change. As a learning healthcare system with the mission of caring for Veterans and their families, patients’ needs are at the forefront of VHA policies and innovations (Kilbourne et al., 2024a, 2024b). VHA prioritizes the integration of Veterans’ care in the organization’s cultural values and practice. Similarly, the VHA benefited from having a wealth of resources that facilitated innovations and adaptations. As participants noted, most programs leveraged VHA’s integrated healthcare networks, existing telehealth capabilities, and social resources to meet patients’ needs. Additionally, sites that were flexible and open to change were more likely to have participants describe adaptations fostered by a learning climate. Conversely, strategies aligned with CFIR elements such as networks and communication were less frequently reported, highlighting the challenges cited by many participants.

Based on our study findings, we identified **three key strategies** that may be useful in future public health crises and rapid organizational changes. These strategies, focused on expanding VHA’s PS capacity to meet patients’ healthcare needs, can also benefit other healthcare systems, which are increasingly incorporating peer support models of care (Center for Mental Health Services, 2024). The first is to ***create an ongoing learning climate***. Having a strong existing work culture that is receptive to change and innovations helped buffer the challenges posed by COVID-19 restrictions and facilitated rapid adaptation in time of need. To illustrate, while all PS had access to VHA resources, PS at sites that had an ongoing learning climate were more successful at identifying and leveraging innovative VHA resources and taking risks to develop new initiatives and shift peer support services. Those who had adopted a culture of learning in their peer support services discussed being flexible and more open to testing new strategies.

Related, the second strategy is to promote ***networking and collaboration***. Participants at sites that actively pursued collaboration observed that it reduced isolation, fostered creativity, and reduced program silos, all of which are critical for maintaining productivity and employee engagement in times of crisis. The third strategy is to ***foster PS self-efficacy.*** Sites that incorporated PS in their adaptation processes noted that they offered new training and leadership opportunities to PS, fostered their sense of autonomy and self-efficacy, and encouraged them to develop new initiatives. Indeed, many of the new initiatives that attended to Veterans’ unmet social needs during the study period were initiated by PS. Moreover, supervisors from these sites discussed empowering their PS to engage in team activities and decision-making processes. They embraced clear communication strategies, such as holding more frequent supervision sessions and maintaining an open-door policy to support PS (Abu Dalal et al., 2022; Montano et al., 2017; Wang et al., 2011). In brief, these results highlight the potential impact of transformational leadership in times of crisis. In this context, transformational leadership inspires innovation and maximizes autonomy for staff to provide high quality care for Veterans with evolving needs. Transformational styles typically foster more open communication, psychological safety, and cohesive team cultures, in addition to staff well-being (e.g., work engagement) (Bass & Avolio, 1994; Corrigan et al., 2002; Montano et al., 2017; Wang et al., 2011).

Notably, PS reported changes in interpersonal and team-wide dynamics that influenced their ability to do their job. Some PS who once enjoyed independence and trust at work reported sudden changes in this treatment, including micromanagement and increased restrictions. This change, which may have been a reaction to productivity demands, left many PS feeling betrayed, which was further amplified by some programs’ lack of transparency in decision-making processes and ineffective team communication. Importantly, social and autonomy support, as opposed to restrictions and micromanaging, have been found to be particularly important for PS performance and job satisfaction (Brooks et al., 2021). In a study of pandemic impacts on a wide range of mental health staff, extensive work changes because of the pandemic increased staff burnout and subsequent turnover intentions, regardless of job resources added by organizational leaders (Sklar et al., 2021). Our study findings further emphasize the importance of attending to team members emotional well-being and team-wide support to buffer burnout during times of crisis.

Moreover, our findings have relevant implications for peer support services across healthcare organizations. Consistent with previous research, PS in VHA point to relationship dynamics and implementation barriers that have impeded PS effectiveness and their full integration in clinical care teams (Chinman et al., 2021; Cooper et al., 2024; Crisanti et al., 2022; Mutschler et al., 2022; Peeples et al., 2023). The findings also highlight opportunities for ongoing reflections, assessment, and quality improvements to support the implementation of peer support services.

Our study also demonstrates PS agility, creativity, resilience, and high potential to make meaningful contributions to VHA as a learning health system and to Veterans’ mental health in a time of rapid change and increased need for mental health services. PS were actively engaged in promoting public health. Because they were often the first point-of-contact for many Veterans and had more flexibility in their schedule, they were well-poised to promote Center for Disease Control guidelines for COVID-19 infection risk reduction. They also served as role models in educating and supporting Veterans to practice effective self-care, chronic disease management, and to maintain engagement in health services. Importantly, they also provided important emotional support at a time when many Veterans were experiencing heightened emotional distress. Moreover, PS used their shared lived experiences, social identities, and contexts as Veteran patients to provide valuable insights that shaped patient-clinician interactions and clinical services. In brief, VHA PS consist of a valuable, well-trained, and community engaged workforce that can efficiently assist in supporting public health promotion, mass health communication, and access to health services in future health crises (Fisher et al., 2018; Litzelman et al., 2022; Wright-Berryman et al., 2011).

Our findings are based on diverse, in-depth perspectives of PS and supervisors from multiple VHA facilities. Though these results are an important addition to the literature, they must be interpreted with caution as they are limited to one VISN and region of the US and may not reflect the current state of VHA peer support services. Further, the VHA is the largest integrated healthcare system in the U.S. with unique characteristics that benefit patient care compared to other health systems. For example, VHA’s primary mission is to provide healthcare to Veterans, allowing the system to be tailored to unique Veterans’ needs and it is funded through congressional appropriations. It has centralized data systems, coordinated resources, and a history of rapid deployment of best practices, including telehealth services. In brief, PS and their supervisors in the VHA had access to many resources that helped them adapt to meet Veterans’ needs – resources that may not be available in other healthcare settings.

Additionally, the interviews were conducted during the early period of the COVID-19 pandemic. The challenges discussed are specific to the study period and do not reflect the current state of VHA peer support services. However, they can serve as a reference point for understanding the development and evolution of these services over time. Given the potential for future health crises and rapid organizational changes, we also sought to identify strategies that facilitated peer support services adaptations, with the goal of informing future preparedness efforts and contributing to ongoing improvements in health services delivery. Since the time of this study, the VHA Office of Mental Health’s Peer Support Services Section has updated the scope of practice and national standards of practice for PS and have expanded educational and training resources for PS and supervisors (Department of Veterans Affairs, 2025). These changes, along with ongoing efforts from the VHA Office of Mental Health’s Peer Support Services Section, may help further facilitate the implementation of peer support services in VHA and enhance responses during times of crisis. However, additional research is needed to evaluate their effectiveness and sustainability.

## Conclusion

The results indicate that, during times of crisis, foundational organizational practices, such as change management techniques and transformational leadership styles, can help support PS and improve peer support services delivery to Veterans. Lessons learned during the pandemic may be applicable in our current time of policy uncertainty, and rapid changes that may occur at any time – budgetary, administrative (i.e., new electronic health records implementation, return to office), and policy changes – and that may upend healthcare services delivery. Rapid organizational changes could amplify negative impacts on team members with less power. Therefore, careful advance planning as well as ongoing monitoring may be needed to ensure that all teams members feel engaged, supported, valued, and included.

## Data Availability

Upon request, a limited dataset will be made available to other researchers for the purposes of validation of findings. The limited dataset will include de-identified data relevant to the specific request and with the permission of the U.S. Veterans Health Administration.
